# Recent Advancements in Polythiophene-Based Materials and their Biomedical, Geno Sensor and DNA Detection

**DOI:** 10.3390/ijms22136850

**Published:** 2021-06-25

**Authors:** Seyyed Mojtaba Mousavi, Seyyed Alireza Hashemi, Sonia Bahrani, Khadije Yousefi, Gity Behbudi, Aziz Babapoor, Navid Omidifar, Chin Wei Lai, Ahmad Gholami, Wei-Hung Chiang

**Affiliations:** 1 Department of Chemical Engineering, National Taiwan University of Science and Technology, Taipei City 10617, Taiwan; mousavi.nano@gmail.com; 2 Nanomaterials and Polymer Nanocomposites Laboratory, School of Engineering, University of British Columbia, Kelowna, BC V1V 1V7, Canada; sa_hashemi@sums.ac.ir; 3 Department of Medical Nanotechnology, School of Advanced Medical Sciences and Technologies, Shiraz University of Medical Sciences, Shiraz 71345-1583, Iran; S.bahrani22@gmail.com; 4 Department of Materials Science and Engineering, School of Engineering, Shiraz University, Shiraz 71348-51154, Iran; khadije.yousefi@gmail.com; 5 Department of Chemical Engineering, University of Mohaghegh Ardabili, Ardabil P.O. Box 179, Iran; gitybh@gmail.com (G.B.); Babapoor@uma.ac.ir (A.B.); 6 Department of Pathology, Shiraz University of Medical Sciences, Shiraz 71345-1583, Iran; 7 Nanotechnology & Catalysis Research Centre (NANOCAT), University of Malaya (UM), Kuala Lumpur 50603, Malaysia; cwlai@um.edu.my; 8 Biotechnology Research Center, Shiraz University of Medical Sciences, Shiraz 71345-1583, Iran; gholami@sums.ac.ir

**Keywords:** polymers, biochemistry, antibacterial, conductive polymers, biosensors

## Abstract

In this review, the unique properties of intrinsically conducting polymer (ICP) in biomedical engineering fields are summarized. Polythiophene and its valuable derivatives are known as potent materials that can broadly be applied in biosensors, DNA, and gene delivery applications. Moreover, this material plays a basic role in curing and promoting anti-HIV drugs. Some of the thiophene’s derivatives were chosen for different experiments and investigations to study their behavior and effects while binding with different materials and establishing new compounds. Many methods were considered for electrode coating and the conversion of thiophene to different monomers to improve their functions and to use them for a new generation of novel medical usages. It is believed that polythiophenes and their derivatives can be used in the future as a substitute for many old-fashioned ways of creating chemical biosensors polymeric materials and also drugs with lower side effects yet having a more effective response. It can be noted that syncing biochemistry with biomedical engineering will lead to a new generation of science, especially one that involves high-efficiency polymers. Therefore, since polythiophene can be customized with many derivatives, some of the novel combinations are covered in this review.

## 1. Introduction

Electronic conductive polymers have garnered enormous interest in drug delivery and biomedical applications [1,2,3]. Recently, some ICPs, such as polypyrrole (PPy), polyaniline (PANI), and polythiophene (PTh), have been widely applied in different applications, such as electromagnetic-interference shielding, chemical sensors [4], rechargeable batteries [5], photovoltaic cells [6], bio-molecular immobilized matrices [7], membrane separation [8], corrosion tools, and microwave adsorption [9]. ICP is a novel descendant of new polymeric compounds that have delocalized electronic structures which are sensitive to variations of the environmental polymeric chains and other disturbances in the chain structure. A drawback of the functionalized polymers with the post-polymerization process is the potentially detrimental effects of the polymer films being adhesive to the surface of the electrode, which is important for biosensing [10]. Conducting polymers (CPs) were first introduced during the mid-70s, and they were deemed as a novel production of organic substances which had both electrical and photosensitive properties, similar to inorganic semiconductors and several metals. In addition, CPs also have some satisfactory properties, such as simple synthesis pathways and flexibility in processing.

The detection and determination of certain DNA sequences is a novel usage in several applications, including clinical diagnosis and medical research. Hence, electrochemical Geno sensors are widely utilized due to their great selectivity and acceptable sensitivity. Some factors, such as satisfactory hydrophobicity, suitable surface area, and high cell specificity, are required for neural probe materials to develop and maintain a quality signal-to-noise (S/N) ratio [11]. The polymers of the poly (alkyl thiophene) family have previously been used because of their high conductivity and their favorable orbital overlapping along the backbone of the polymers [12]. Recent research has also illustrated that these polymers could be modified with an extensive spectrum of functional ending groups, such as one that allowed metal self-assembly [12]. However, medicinal analysis may face a shortage of supplies in the case of emergencies. Measuring the levels of a drug is the cornerstone for drug discovery in biological fluids and also for pharmacodynamics, pharmacokinetic investigations, and drug detection [13]. Using grafted PTh is also well known to boost the carrying of DDS through the cell membrane using endosomal membrane hydrophobicity and rigidity [14].

The discovery of novel pathways to develop the platforms for monitoring DNA with high sensitivity and selectivity is still a challenging issue and needs more attention, especially in biomedical uses and medical detections. Recently, methods utilizing connected polymers to transmit combination events to an electronic signal were recorded [15]. In some cases, this recognition is related to the modification of electrical features of a functionalized conjugated polymer by using oligonucleotide and then combining it with its potent desired nucleic acid [16]. The major concerns of such applications are a low value of S/N ratio due to the continuous presence of adsorbed polymer on the substrate and also the reduction in electrical signal following certain target recognition [17]. Cationic PTh includes special abilities to recognize DNA in complex aggregations [18]. Furthermore, cationic polymers are favored as they can be assembled quickly with DNA via electrostatic interaction to achieve complexes of polyelectrolyte. Such an approach is affected by various factors, including pH, ionic strength, and temperature. These parameters influence the polyanion/polycation interactions and are commonly well-known. The variations of such factors can also be utilized to improve dissociation for the on-demand assembly of polyelectrolyte [19,20].

## 2. Conjugated Polythiophene (PTh)

Conjugated PThs and their valuable derivatives are introduced as a novel member of the conjugated polymer group due to their unique characteristics, such as electrical behavior, thermal stability, high environmental behavior, mechanical robustness, and cost-effective preparation [9]. A wide range of modified thiophenes had been prepared in the past for their needed features. Usually, diverse thiophenes with interesting functional groups that can immobilize biocompounds (for instance, eCOOH and eNH_2_) are hardly electropolymerized, as their functional groups demonstrate vital nucleophilicity, which proves that the radical cationic intermediates create the ithin electropolymerization process, and thus resulting in the harming of the polymerization process [21].

In addition, the electro polymerization of 3-thiopheneacetic acid has been reported [22], in which a great oxidizing potential (w1.6 V vs. Ag/Agþ) and great value of monomers were required to obtain a conductive polymer. On the other hand, at a low value of monomers, the electro-oxidation and homo-polymerization of thiophene-acetate were inhibited [23]. The issue is solved by post-polymerization functionalization as the monomers of thiophene can be simply electropolymerized along with the protective carboxylic acid groups. This method is a new approach to prepare novel electroactive organic platforms with well-proportioned electronic, spectroscopic, and electrochemical features [24].

It is believed that thiophene can be combined with a variety of materials, mainly polymers with conjugated nanoparticles, to enhance its stability and required performance [25,26,27,28]. Developing organic biocomposites for tissue engineering is considered to have a bright future in medical fields. A vast range of materials is known to be beneficial when combined with thiophene, such as nano clay, for improving the antimicrobial effects [29], compounds prepared by nanocomposites to facilitate the delivery of drugs [30], nanofibers for managing the drug release systems [31], carbon Nanotube [32], silica [33,34], epoxy [35] and, etc.

## 3. Electrode Coating

An extensive range of approaches has been reported by researchers where the surfaces of the electrode were coated with soft biomolecules layers, such as layer-by-layer assembly, hydrogels, and multi-layer assembling [36]. These coating approaches enhance the separation of the target neuron and electrode, and they can also enhance the independence of electrodes. Both of these impacts can reduce the strength of the neural signal and therefore lead to worsening of the in vivo performance. Using the mixed films of some conductive polymers and biomolecules can be a beneficial pathway for electrode coating development. Indeed, these proposed films provide lower electrode impedance and higher acceptable biocompatibility [37].

It is a noticeable fact that almost all coatings with conducting polymer for neural probes are built by electro-co-deposition of biocompatible anions and polymer at particular sites of the electrode. Such a process enables the properties of films that are tailored for each electrode. However, it can be noted that the electrodeposition route does not allow specific parts to be controlled; for example, the relative amounts of biomolecule and polymer in the film or 3-dimensional display of such components are at the micro-/nano-meter diameters. Furthermore, films are just bonded on the surface of the electrode via electrostatic interactions owing to mechanical interactions or decreasing the attack of metal/polymer interface by delamination of electrolyte [38]. It is suitable to build a conductive polymer that is strongly connected with the electrode, which will enable the composition of the film to be controlled. In contrast, by using some nanomaterials, such as epoxy [39] and nanotubes [40], as the potent filler for thiophene composites, a higher total electrical conductivity can be achieved.

## 4. Self-Assembled Monolayers (SAMs)

**Figure 1:**
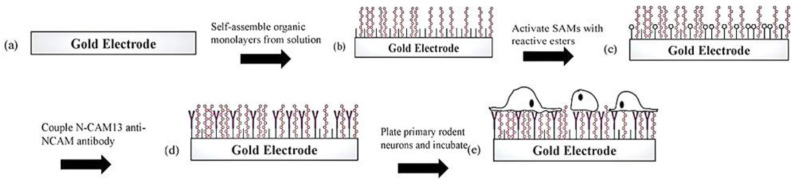
Assembly schematic for SAM-based neuron/electrode interface (**a**,**b**) Formation of mixed EHPT/MHA SAM by incubation in organic thiol solution; (**c**) formation of reactive NHS esters on MHA carboxyl groups; (**d**) capture of adhesion-promoting biomolecules (N-CAM13 anti-NCAM) by NHS ester reaction with free amino groups; (**e**) quenching of unreacted esters and plating of primary neurons that bind to the coupled adhesion molecules [44].

SAMs are linked covalently to substrates, and they create monolayers on the surface [41]. Generating well-loaded SAMs can usually be achieved via substrate incubation in an organic diluting solution of assembled molecules at nighttime. Once they are created, SAMs consist of covalent coupling surface groups for biomolecules [42]. SAMs are often strong enough under different biological situations, and their attachment to the substrate can be regenerated through electrochemical functions [43]. These layers can be described in three distinct sections. First, the PTh’s ability is verified to form SAMs through the AFM test. Next, SAMs are modified with cohesive neuronal compounds and then cultured with initial neurons of mouse to indicate their biocompatibility. Finally, the SAMs’ electrical impedance is measured with and without combined adhesion compounds in order to reveal the functionality of these coatings for the improvement in the characterizations of electrodes. These coatings are capable of enhancing the electrodes’ properties. All SAMs are shaped by using thiol ‘1 groups’ interactions to clean gold surfaces. Figure 1 illustrates the schematic process of SAM formation through cell culture. 

**Figure 2:**
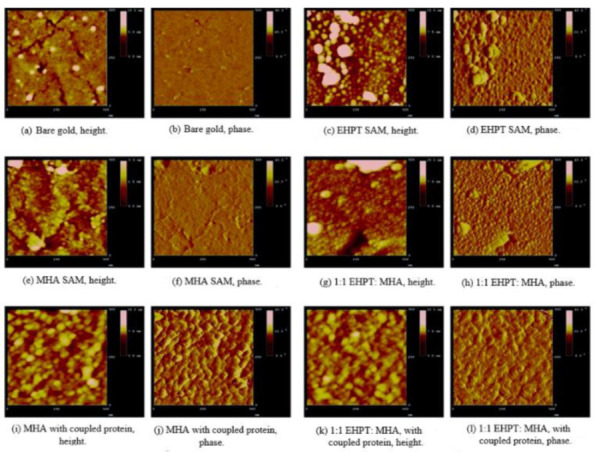
Atomic force microscopy of pure and mixed EHPT and MHA SAMs on template-stripped Au (111). All images were acquired in tapping mode with a scan speed of 1 Hz. (**a**,**b**) Bare gold. Wide and flat terraces with smaller subgrains are seen; the tall specks are presumed to be dust particles adsorbed to the surface. (**c**,**d**) EHPT SAM. The underlying terraces are concealed by this thick, granular film. (**e**,**f**) MHA SAM. This tightly packed thin organic monolayer essentially mirrors the underlying terrace structure. (**g**,**h**) Mixed 1:1 EHPT:MHA SAM. This strongly resembles an EHPT SAM, with polymer granules seen across the entire surface. Despite the EHPT, the underlying gold is not entirely effaced. (**i**,**j**) MHA SAM after IgG coupling. Large globular protein molecules (likely including small aggregates) cover the surface. (**k**,**l**) Mixed 1:1 EHPT:MHA SAM after protein coupling. The surface now matches the MHA/protein image, showing the same irregularly globular structures [44].

**Figure 3:**
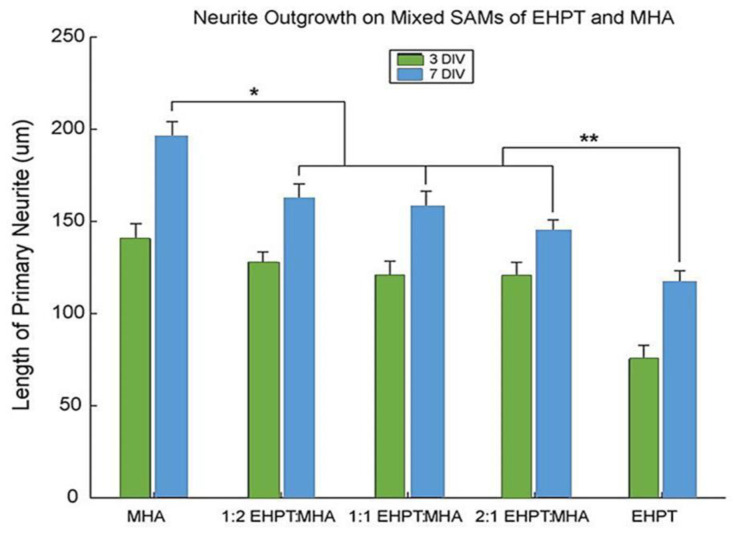
Outgrowth of neurites on pure and mixed EHPT/MHA SAMs at 3 and 7 DIV. Average length of each cell’s primary (longest) neurite is reported. At 3 DIV, only the EHPT group differs significantly from the others (*p* < 4.624 × 10^−5^). By 7 DIV, pure MHA shows significantly greater outgrowth than all mixed SAMs (*p* < 0.0087, denoted by *), and pure EHPT (with protein adsorbed) shows significantly less growth (*p* < 0.0233, denoted by **). There is no significant difference at 3 or 7 DIV among the three mixed SAMs tested (*p* > 0.3287) [44].

During the tapping state of the AFM analysis of the net and mix SAMs of MHA and EHPT with a blank template/stripped Au, pure SAMs were formed from MHA, as shown in Figure 2e,f. They were slightly different from the bare TSG. As seen from the results of these scans, single MHA molecules were way smaller compared to the cantilever tip and must be created in a high-loaded monolayer, which reflected the underlying topography. The presence of MHA can only be checked through its capability to take part in coupling reactions of protein, as observed in Figure 2i,j. Following the reaction which happens to couple the materials, the height across the surface changes while the underlying Au structure is unclear. Figure 2g demonstrates the performance of SAM configuration of a complex solution. The figure indicates the features of EHPT and MHA net SAMs and also the more desirable EHPT. The granules, which were approximately 10–15 nanometers tall, can be observed to be similar to those in EHPT; however, some cracks in gold terraces were still observed. As indicated in Figure 2k,l, by coupling the proteins, the surface had modified the pure MHA SAM. Despite considering the condition that the top SAMs were maintained in ambient temperature (without the presence of light) over the course of time (days or even months), any important variations over time in the images of SAM were apperceived (data not represented). As illustrated in Figure 3, SAMs, EHPT, and MHA compounds are at an appropriate neuro compatible scale and are able to maintain the continuation of neurite outgrowth up to 7 DIV. The quantitative efficiency of mixing SAMs among pure SAMs of each component is noticeably distinct. While the neurons’ longer-range durations of SAMs have not been reported, it can be assumed from prior experiences with the primary neuron cultures that a culture that remains until 7 DIV can remain perpetually as high as the mean, which is regularly varied. 

The influence of coupling MHA with NCAM13 is a result of a certain linkage of NCAM13 to NCAM over the cultured nerves; hence, it is believed that the proteins do not form permissive substrates while combined with anything other than the adhesion compounds (such as albumin). It is interesting to report that there was no considerable difference among any of the mixed tested SAMs. Nonetheless, this can be clarified based on the powerful impact observed through the AFM analysis. If more surface area is needed, it is predicted that the obtained 55% impedance will be reduced just after the formation of EHPT SAM. Some researchers have illustrated the electrochemical rise in PThs, such as both syntheses from the covalently linked self-assembled initiators and preparation routes [45]. Even though such methods are complex because of the ability of PThs to be overoxidized at potentials close to the potential of their synthesis, they can be embraced to enable the creation of films that are significantly rougher and thicker while still being linked chemically to substrate.

## 5. Polythiophene in the Detection of Adrenolytic Using SPME-HPLC

The SPME-HPLC/UV method is a sensitive and easy way to determine the adrenolytic drug therapies which are characterized by small biological volumes in major diseases, including chronic atrial fibrillation (AFib) or hypertension, which requires direct blood monitoring. This method is known to be able to evaluate the blood levels in adrenolytic drugs and is an effective device for extracting them selectively in clinical analysis. Hence, polythiophene and polypyrrole had been chosen as the main adsorbent in solid-phase microextraction sampling of five adrenolytic drugs in the human plasma models. The SPME coatings of polythiophene and polypyrrole affect the extraction capacity in terms of sensitivity and selectivity with respect to the desired molecules. Therefore, in order to define the beta-blockers in the mentioned models, using PPy and PTh can be very beneficial [46].

The results of the extraction using polythiophene fibers indicated that the polythiophene fibers worked perfectly at the desorption processing pH of 7.0 for the drugs oxprenolol and metoprolol and in pH of 8.6 for the drugs propranolol, mexiletine, and propaphenon. It is necessary to note that both PPy and PTh fibers that were obtained using the electrochemical polymerization method can act as an adsorbent for microextraction (solid-state). Fifteen grams per milliliter was the optimum concentration for each experimental drug. The functionality of PPy and PTh in the human plasma models was assessed via six different concentrations with descending quantity of adrenolytic drugs (30 g/mL to 5 g/mL). Adsorption and desorption time was calculated at periods of 10 and 5 min, respectively. The efficacy of each tested drug in the applied adsorbent material is described in Table 1. Comparison of the extraction results between PPy-SPME and PTh-SPME fibers from the human plasma models for adrenolytic drugs at 5 g/mL concentration indicated that the pH of 8.6 demonstrated the ideal outcomes, while metoprolol displayed the greatest extraction functionality from all the investigated adrenolytic drugs. In the end, it was indicated that the PThSPME fibers showed better extraction potentials compared to PPySPME fibers [46].

## 6. Molecular Gate

In a recent study, the linkage copolymerization of hydrophilic methyl methacrylate monomer and N,N-dimethyl aminoethyl methacrylate on PTh through the ATRP method was studied for molecular gates. Furthermore, PTh-*g*-poly (di-methylamino ethylmethacrylate) (PTh-*g*-PDMA) was synthesized [47,48]. The PL-intensity of the PTh-*g*-PDMA solution decreases as the temperature increases at the pH of 7 as a result of the collapse of the chains of PDMA onto the PTh core, which shows that the intensity of PL is enhanced in the MC gel as a compensation of the aforementioned reduction. Based on such results, it was proposed that the prepared PTh-*g*-PDMA graft copolymer could act as an AND fluorescent molecular logic gate type system with fluorescence as the output and pH and temperature as the inputs. The highest sensitivity of the logic gate was pH-dependent, and it occurred at a high pH (pH 9.2) rather than at other conditions, such as pH = 4 at the temperature of 45 °C. Such results are ascribed to a variation in polarity of the micro-environment of the PTh chains related to PTh-*g*-PDMA within the methylcellulose gel, particularly because of the variation of pH and temperature [9].

## 7. DNA Hybridization Electrochemical Biosensor

**Figure 4:**
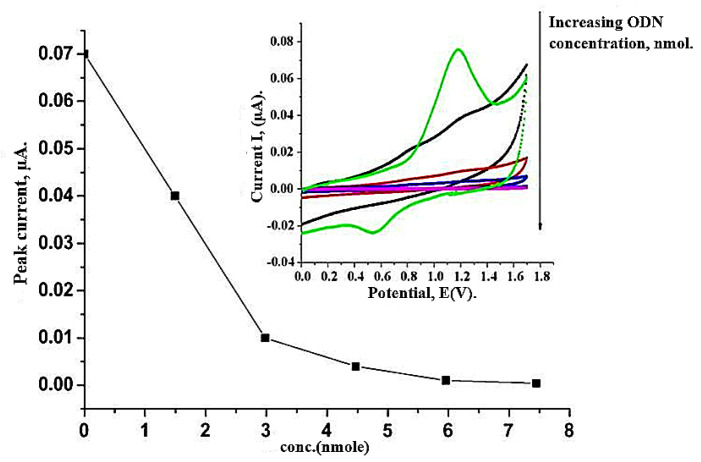
Variation of electrode current at constant potential *E* = 1.2 V, as a function of concentration of target ODN (inset: cyclic voltammograms of PHPT/ODN bioelectrode with increasing target ODN concentration, mmol) [49].

Due to the sensitivity of the biosensor, various values of ODN, targeted in the range between 1.49 and 7.45 nmol, were measured using electrochemical approaches. Figure 4 shows the CVs of bioelectrode modified with poly(hydroxyl phenylthiophene-carboxylate, PHPT)-ODN by enhancing the value of the ODN target and the change in anodic peak signal versus the value of the physisorbed ODN on PHPT film via the CV method. It can be observed that the oxidation signal at 1.2 V was reduced when the value of the ODN target increased. This outcome shows the further improvement in selectivity and sensitivity achieved via PHPT nanocomposites. An unlabeled and simple electrochemical sensor was prepared to detect the DNA event using electro polymerization of hydroxyphenyl-thiophenecarboxylate. PHPT was synthesized on GCE using the electrochemical methods, and its characterization can be traced through CV, AFM, and FTIR analyses. The probe of ODN was physically adsorbed onto the PHPT film and assayed on a combination of the supplementary parts of ODN. A bio-recognition can be carried out by comparing the electrochemical currents (CV analysis) of the single-stranded and double-stranded oligonucleotide. The anodic peak signal current of the single strand was higher than the one in dss-oligonucleotide, which was related to the reduction in the PHPT electroactivity and the enhancement of the rigidity of the structure of polymer. The Physisorbed probe of ODN and its combination were revealed morphologically on ITO electrodes through the AFM. The electrochemical sensor sensitivity and detection limit were 0.02 µA/nmol and 1.49 nmol, respectively, with excellent selectivity.

The HPT can be electropolymerized within the electrolyte TBAClO_4_/acetonitrile to the respective PHPT. The anodic oxidizing peak was achieved at 1.2V [49]. With increasing film thickness, the polymer color varied from brown color to dark green-red color. Contrary to alkyl-substituted PTh [50], PHPT is insoluble in the usual organic-based solvents, including chloroform and acetonitrile. Following several repetitions of scans, the modified electrode was rinsed completely with acetonitrile solvent to eliminate oligomeric and monomer species. Then, the prepared electrode was placed in a free electrolytic-monomer media with 5 mM TBAClO_4_/acetonitrile. The polymer film showed a great electroactivity in that media with a relatively reversible anodic peak at 1.2 V and a linearly current dependency of CV on the scan rate. The responses of the oxidizing peak were estimated, and it was shown that the oxidation signal linearly increased as the scan rate increased. It was obvious that the redox behavior was not diffusional, and the electroactive polymer was excellently attached to the surface of the electrode [51]. It can be observed that the current intensities of the probe of ODN and the immobilized ODN electrodes had reduced, which can be ascribed to the advanced redox activity of the probe of ODN [51].

## 8. Electrochemical DNA Hybridization Detection

In the present research, the PTh modified with MB (PMT/MB) was prepared, and its feasibility as a marker for electrochemical detection of DNA was surveyed. The current variation of PMT-MB for supplementary ODN is four times higher than the non-supplementary ODN with a similar value. There was a linear relationship between the variation of current and the logarithmic values of ODN in the range of 6.37 nM to 0.204 µM. The PMT-MB signal response to non-supplementary ODN showed excellent selectivity towards the recommended identification tool. In addition, as the electrochemical marker, PMT-MB exhibited high resistance against variations in values of the electrolyte (changing in ionic strength). For all tested concentrations of electrolyte, the combination always led to the enhancement of the PMT-MB signal due to the electrostatic interaction between dsODNs and PMT-MB. On the other hand, even though MB was applied as a control material, the combination led to a large reduction in the current of the applied electrolyte solution and a small increase in the current owing to the variation in the dominated interaction. The interaction between dsODNs and PMTMB was investigated further via UV-Vis spectrometric methods. For dsODNs with low value, the PMT-MB main binding mode with dsODNs was intercalation, and for dsODNs with great value, the main binding mode was electrostatic interaction. The outcomes demonstrated that PMT-MB can selectively and effectively identify supplementary targets of ODN as an electrochemical indicator and possessed a high capability to be used in life science and medicinal diagnostics.

3-(3-Bromopropoxy)-4-methylthiophene (BMT) was synthesized based on the recorded methods [52]. Following polymerization, FeCl_3_ was employed as an oxidizing material, whereby the obtained polymer PBMT had a reaction with the methylene-blue in N-methyl pyrrolidone within a 5-day period in a dark media at ambient temperature. Subsequently, the PMT-MB was precipitated by adding acetone, which was saturated with tetra-butyl ammonium chloride. The PMT-MB product was purified using the acetone solution and the soxhlet extracting method. To assay the performance of the PMT-MB as an electro-indicator for combination recognition, an ODN probe with modified Au-electrode was incubated in the ODN target media. The identification of various ODN targets was performed by gathering DPV peaks of PMT-MB on the surface of the electrode in the blank PBS.

**Figure 5:**
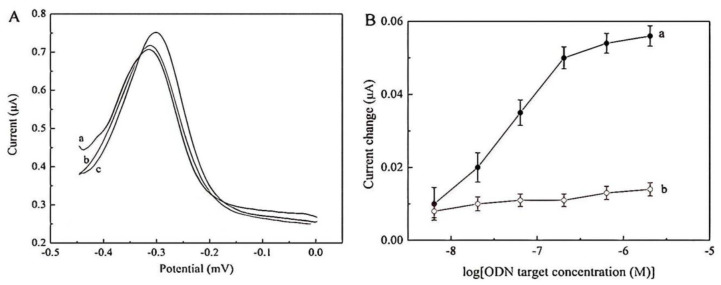
(**A**) Differential pulse voltammograms of accumulated PMT-MB on modified gold electrodes. (a): ssODN modified; (b): after incubation with 0.204 _M of noncomplementary ODN target; (c) after incubation with 0.204_M of complementary ODN target. (**B**) The current change in DPV response of PMT-MB with the concentration of complementary ODN target (a) and non-complementary ODN (b). PMT-MB was accumulated for 25 min in 5 mM phosphate buffer solution containing 0.032 mg/mL of PMT-MB. DPV was measured in blank phosphate buffer [53].

Figure 5A shows the DPV plots of PMTMB accumulated on the functionalized electrode via ODN probe before and after incubation in the ODN solutions. Following the incubation in 0.204 µM of non-supplementary ODN, a small increase in signal was observed (Figure 5A (b)), which may be due to the adsorbed non-specific non-supplementary target, which led to the increased value of accumulated PMT-MB on the surface of the electrode. On the contrary, following the incubation with the supplementary target of ODN, the peak of the current increased greatly (Figure 5A curve c), which demonstrated that there was more accumulated PMT-MB on the surface of the electrode owing to the ODN duplex formation. This outcome clearly revealed that the PMT-MB can be applied as an efficient indicator for electro-recognition of ODN hybridization [53].

## 9. Specific and Sensitive Detection of DNA by PTh

The strategy of amplification according to the application of electroactive aggregates and the considerable sensitivity of the absorptive stripping SWV was considered. The specific and sensitive electrochemical recognition obtained was as low as the zeptomoles of the ss-DNA (about 2500 copies into the 10 μL). This method used the specific hybridization of a wide range of electroactive moieties, which recorded each combination case due to the polymeric nature of the transducer and the good DNA recognition abilities of the cationic PThs in the form of aggregation. Therefore, the electrochemical recognition techniques were incorporated into the combination of PNA features with respect to DN. A novel electrochemical amplifying approach is employing water-soluble aggregates for ultrasensitive recognition of DNA, as ferrocene modified PTh, was also analyzed. The method included a quantitative, sandwich-type of method that targeted the DNA which was absorbed by the PNA probes, and the aggregations of polymer/DNA acted as the transducers. The electrochemical recognition was performed on an adjacent bare electrode where the resulting aggregates were separated from the array of PNA and fragmented. A pre-concentration approach of ferrocenyl groups was carried out by stripping the SWV to further enhance the performance of the sensor. Such an approach shows the novel hybridization of a large number of electroactive moieties, which records each combination event, due to the polymeric nature of the transducer and its abilities to detect the DNA related to the cationic PThs in the aggregation system. It combines the simplicity of electrochemical methods and their high sensitivity for higher combination affinity of PNA to DNA, thus allowing DNA recognition of about 4 moles LOD (2500 copies in 10 μL) with superior selectivity towards mismatched DNA [19].

## 10. Colorimetric Recognition of RNase H and microRNA Using Cationic Derivatives of PThs

MicroRNAs (miRNAs) are known as non-protein-coding, small-sized RNAs (almost 19 to 23 nucleotides), which have been detected in animals, viruses, and plants. Gene regulation using miRNAs plays an impressive role in a large number of main biological functions, including development, metabolism, differentiation, and immunological response [54]. Recent investigations had demonstrated that the patterns of miRNA interpretation were accompanied by different types of tumors [55]. Therefore, to find out the performances of miRNAs in the detection of diseases and also to find novel drug targets, robust research is being performed worldwide with the aim of discovering effective ways to detect miRNA [56]. Some homogeneous techniques to detect miRNA have also been recorded, including real-time PCR, amplification based on ribozyme, and modified invader assay [57]. Nevertheless, such homogeneous techniques require complex approaches and multiple fluorescence tags, leading to great cost and long analysis time. 

Hence, performing simple, homogeneous, and label-free tests is the challenge in detecting miRNA. RNase H nuclease is a certain endolytic nuclease of RNA in RNA/DNA hybrids. It plays a certain role in some cellular approaches, which include replication, transcription, and repair of DNA [58]. In addition, the RNase H behavior of HIV-1 reverse transcriptase is a novel target for HIV-1 inhibitors as there is a good possibility to improve novel anti-HIV therapeutics [59]. Therefore, the assay of the activity of RNase H has high significance in biological studies and for the screening of novel anti-HIV drugs. Cationic water-soluble poly (N,N,N-triethylamino-propyloxy)-methyl-thiophene hydrochloride) was chosen as the optical probe for miRNA detection. To verify the specificity of the perfectly complementary DNA, the proposed method for miRNA detection, which had one or three bases of DNA mismatched with the non-complementary DNA, were combined with miRNA, followed by the addition of the 3-Unit RNase H into the solution of miRNA/DNA for the digestion of miRNA. Thereafter, the solution was combined with PMNT. 

**Figure 6:**
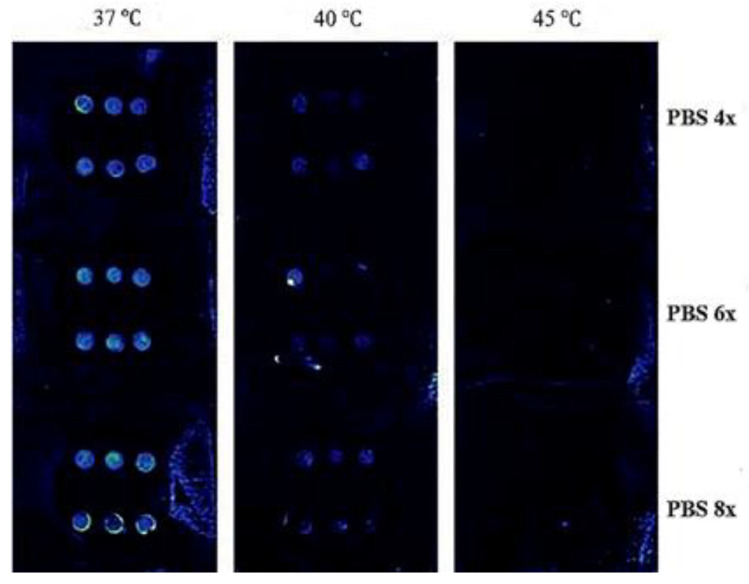
Inverted micrographs of HeLa cells grown in the absence and presence of the oligosiloxane modified thiophenes. Panels A and D are control after 18 h and 7 days, respectively. Panels B and E are poly(VI)-co-poly(3-methylthiophene) (50/50, *w*/*w*), after 18 h and 7 days, respectively. Panels C and F are poly(VII)-co-poly(3-methylthiophene) (50/50, *w*/*w*), after 18 h and 7 days, respectively.

The absorbance ratio ranging from 400 nm to 525 nm was used to represent the color change (A525 nm/A400 nm). The results (Figure 6) indicated that a well-specified signal was recognized from the combination of the complementary miRNA/DNA. For the case of three-base and one-base mismatched miRNA/DNA, the A525 nm/A400 nm was 42% and 79%, respectively, in comparison with the complete combination. Hence, with the RNase H digestion tests, the one-base can be discriminated from the whole complementary miRNA/DNA, thus confirming the great specificity of the suggested test. Although miRNA was combined with the non-complementary DNA, there was no miRNA digestion in the presence of RNase H due to the fact that there was no combination between non-complementary DNA and miRNA, and the DNA/PMNT duplexes were created in the media. The creation of PMNT/miRNA duplexes led to the enhancement of the absorbance at 400 nm. In consequence, the lowest A525 nm/A400 nm ratio can be achieved in the case of miRNA and non-complementary DNA. The outcomes confirm that the absorption process and the variation in color changes of the PMNT conformation can be used for the recognition of RNase H behavior with fast routes and a facile tool [60].

## 11. Antibacterial Activity of Thiophenes

It had been proven that some derivatives of thiophene, such as Cefoxitin and Cephalothin, possess satisfactory antibacterial/antifungal attributes against pathogenic bacteria. Ticonazole and Sertaconazole, which have the heterocycle of thiophene, are also known as antifungal agents. Some experiments to synthesize new substitute thiophenes were done using the Gewald reaction to determine their antimicrobial activity. [61]. The various derivatives of parent compound I was obtained by applying various aryl aldehydes to get a group of Schiff Bases (IIa- k), such as R= OH, Cl, CH3, OCH3, NO2, etc. 

The antimicrobial (antibacterial and antifungal) effects of newly synthesized compounds were validated by using spectral data and screened using the disk diffusion method. Monitoring of the reactions was done using layer chromatography on pre-coated plates (SD fine Chem. Ltd.) with various solvent devices. By using TLC, purification of the compound was specified, and iodine vapors were used as visualizing agents. The compound structure was evaluated by using I.R., Mass spectra, and NMR. The antimicrobial behavior was measured using the agar diffusion method at a concentration of 50 mg/0.1 mL where DMSO was used as a solvent [62].

The researchers claimed that the types of electron withdrawal and the process of electron donation were the two major reasons for the high antibacterial activity. Apart from that, it had also been proven that aldehydic phenyl can be considered as a promising ring for antibacterial effects. IId with 2-nitro substitution and IIf with 2-chloro substitution were determined as the most powerful compounds with acceptable performances. Other compounds had demonstrated moderate effects against microorganisms in comparison with their control column. In terms of antifungal performance, the tested compounds demonstrated moderate effect when exposed to *Aspergillus niger.* On the contrary, the antifungal performance of the aforementioned compounds against *Candida albicans* was not acceptable [63,64].

## 12. Anti-HIV Effects of Thiophene

It is believed that the mutations in viral reverse transcriptase can decrease the impression of the first production of inhibitors with no nucleoside-reverse transcriptase (NNRTIs), such as efavirenz (EFV), nevirapine (NVP), and delavirdine (DLV) [65]. It is necessary to note that the emergence of DVL and NVP is the main parameter in reducing susceptibility. Indeed, E138K and K103N are known as the major ones among the existing mutations [66]. Hence, finding and improving new NNRTIs with good efficacy against resistant mutations can be a controversial subject in the field of biomedical chemistry [67].

As reported in the previous investigations [68,69], thiophene pyrimidine [68] can be a satisfactory candidate in the area of diarylpyrimidine (DAPY)-type anti-HIV drugs, which possessed acceptable performance against HIV [68]. Based on their results, the anti-HIV performance and even the SAR assessment of the nine thiophene derivatives were completely promising. Furthermore, a new type of thiophene derivative, which was called (thiophene(3,2-d)pyrimidine), was synthesized in the green pathway, and its potent anti-HIV performance was demonstrated. 

All desired materials demonstrated modest to high potential against WT HIV-1 based on the values of EC50 ranging from 0.0071 μM to 0.196 μM, in which the two most powerful substances (9d and 9a) were certified to be monomorphic inhibitors (EC50 = 7.1 and 9.2 nM, respectively), which were stronger than AZT. These two compounds also possessed moderate potency against most of the tested mutant strains. Specifically, they demonstrated considerable behavior against K103N (EC50 = 0.032 and 0.070 μM) and E138K (EC50 = 0.035 and 0.045 μM). The molecular simulation revealed and proved that the recently introduced sulfamide group, distinct from its isostere amide, can generate extra interactions along with the remaining amino acid in the intolerant region I of NNIBP. It is believed that these results will be a new interest in the research to design NNRTIs based on thiophene pyrimidine with more inherent behavior against the RT mutant HIV strains. The features of a set of three prepared analogs were examined by functionalizing them with amide and sulfonamide substituents at the right part (interaction to the tolerant region I). The left part (A-rings) and the thiophene (2, 3-d) pyrimidine core (B-ring) were retained. Therefore, the tolerant area I of the linked NNRTI (NNIBP) was explored on the basis of the opinion that the novel H-binding receptors/donors may form H_2_ binds with the remaining amino acid of NNIBP, leading to robust linkage and high capability for variants of the associated resistance.

The 4-amino piperidine part of lead was substituted with 1,2-diaminocyclohexane and subsequently sulfonylation, where the acylation with various sulfonyl and acyl chlorides created the desired materials, bearing substituents with changing size and polar group substitution. Indeed, all the obtained derivatives (thiophene d-pyrimidine) were completely characterized through (1H NMR), (HRMS), and (13C NMR) tests. Moreover, the 4-cell MT cultures infected with WT HIV-1 strain, which was associated with the NNRTI-resistant strains, such as F227L + V106A, L100I, Y181C, E138K, and RES056, were used to assess the antiviral performances of the synthesized compounds.

**Figure 7:**
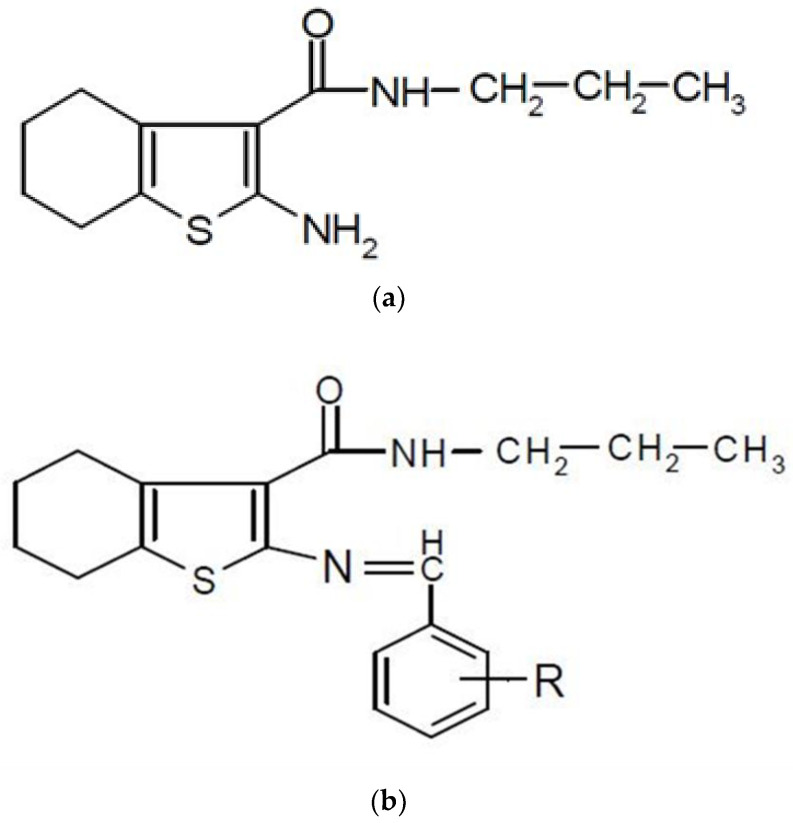
(**a**) 2-((substituted benzylidene) imino)-3-N-(propyl carboxamido)-4,5,6,7-tetra hydro benzo (**b**) thiophenes (Schiffs bases)(IIa-k)): TLC solvent system: Benzene: Ethyl acetate (7:3); Recrystallization solvent: DMF:Water (9:1).

Apart from that, azidothymidine (AZT) and etravirine (ETV) were chosen as the control blank drugs. The obtained results for EC50 (anti-HIV potency), SI (selectivity index, CC50/ EC50 ratio), and CC50 (cytotoxicity) are presented in Table 2 and Table 3. Preliminary SAR analysis demonstrated that the type of functional group (X) in the cyclohexanediamine can significantly affect anti-HIV activity. The compounds of the 9 subseries (X = SO_2_) indicated better activity than those of the 8 subseries (X = CO), i.e., 9b (EC50 = 0.0092 μM) > 8b (EC50 = 0.026 μM); 9c (EC50 = 0.025 μM) > 8c (EC50 = 0.030 μM); 9d (EC50 = 0.0071 μM) > 8d (EC50 = 0.138 μM); 9e (EC50 = 0.086 μM) > 8e (EC50 = 0.196 μM). These results indicated that the tetrahedral structure of the sulfonamide can be more impressive than the planar structure of the amide for hydrogen bonding with the remaining amino acid of NNIBP (Figure 7).

In the 9 subseries, the order of anti-HIV-1 activity was CN > F > Br > CF3, whereas in the 8 subseries, the order was slightly different: F > Br > CN > CF3. Some representative compounds, such as 9a and 9d, were applied to validate the binding target of these newly synthesized thienopyrimidine derivatives. In other words, their performance to prevent the recombination of WT HIV-1 RT enzyme with ETV and NVP again were evaluated as reference drugs. The results illustrated that 9d and 9a demonstrated significant anti-RT capability (IC50 = 1.041 and 1.138 μM), which was superior to NVP but somewhat lesser than ETV. Hence, these novel thienopyrimidine derivatives have great vicinity for WT HIV-1 RT and act as classical NNRTIs [69].

## 13. Conclusions

Thiophene and its valuable derivatives are very effective materials, which can play a vital role in biomedical engineering for usages such as drugs and biosensors. Furthermore, their ability to be flexible in binding many different derivatives is known to be useful for biochemical fields. Therefore, some of the unique electromagnetic polymers, such as polythiophene, were covered in this research for their novel properties with regards to biosensors, gene delivery, and DNA detection. Polythiophene is known to be a good ICP for organic usages. Different electro-conducting methods and many combinations of polythiophene were described above. Moreover, initial results were presented, which demonstrated that polythiophene can be considered as a permanent alternative technology for creating some conductive polymer coatings on neural electrodes. In this review, some items, such as self-assembled monolayers, conjugated PTh and its derivatives, and several approaches for electrode coatings, were summarized in detail. Polythiophene SPME and polypyrrole coating were applied to separate the adrenolytic drugs from human plasma using the SPME-HPLC method. Polythiophene was also applied in diverse gene applications, such as gene sensors and gene delivery systems, with a water-soluble pH-responsive molecular brush of thiophene due to their characteristics and bonding with DNA. They are also a new subject of interest among DNA research, such as electrochemical DNA hybridization detection. Some thiophenes modified with oligosiloxane were used and built for cell growth. These valuable materials can be used in the colorimetric detection of microRNA and RNase applications. A number of compounds were used for identifying the antimicrobial effects of thiophenes to improve the cure and treatment of drug-resistant bacteria. It is hoped that these compounds and experiments will lead to a new generation of medicine and biochemistry science.

## Figures and Tables

**Table 1:** Correlation coefficient and Linearity with relative standard deviations of extraction by PTh-coated SPME fibers from aqueous solutions (*n* = 3) [46].

Parameters	Oxprenolol	Mexiletine	Propranolol	Propaphenon	Metoprolol
Linear range (g/mL)	-	-	-	-	1–150
Slope	146.9	131.65	966.62	1025.5	319.72
Intercept	667.4	465	830.9	4212.2	1054
R^2^	0.9605	0.9644	0.9773	0.9879	0.9856
RSD	1.2	1.1	1.1	1.8	0.9

**Table 2:** Activity and Cytotoxicity against HIV-1 (IIIB) and HIV-2 (ROD) Strains in MT-4 Cells [69].

					EC_50_ (μM) ^a^	
comp	X	SI ^c^ IIIB	ROD	R	IIB	CC50 (μM) ^b^
8a	CO	294	>3.930	H	0.013 ± 0.007	3.930 ± 0.662
8b	CO	121	>3.182	4-F	0.026 ± 0.002	3.182 ± 1.346
8c	CO	93	>2.849	4-Br	0.030 ± 0.005	2.849 ± 1.225
8d	CO	19	>2.617	4-CN	0.138 ± 0.018	2.617 ± 1.655
8e	CO	17	>3.241	3-CF_3_	0.196 ± 0.086	3.241 ± 0.671
9a	SO_2_	340	>3.734	4-NHCOCH_3_	0.010 ± 0.008	3.734 ± 0.157
9b	SO_2_	383	>3.527	4-F	0.0092 ± 0.001	3.527 ± 0.372
9c	SO_2_	231	>5.861	4-Br	0.025 ± 0.006	5.861 ± 3.624
9d	SO_2_	1308	>9.287	4-CN	0.0071 ± 0.0005	9.287 ± 6.187
9e	SO_2_	78	>6.683	3-CF_3_	0.086 ± 0.029	6.683 ± 3.436
ETV	-	776	-	-	0.0028 ± 0.0002	2.18 ± 0.029
AZT	-	>664	0.008 ± 0.001	-	0.011 ± 0.005	>7.484

^a^ EC50: concentration of compound required to achieve 50% protection of MT-4 cell cultures against HIV-1-induced cytotoxicity, as determined by the MTT method. ^b^ CC50: concentration required to reduce the viability of mock-infected cell cultures by 50%, as determined by the MTT method. ^c^ SI: selectivity index, the ratio of CC50/EC50.

**Table 3:** Anti-HIV-1 Activity against Mutant Strains in MT-4 Cells [69].

	L100I	K103N	Y181C	Y188L	E138K	F227L + V106A	RES056
8a	>3.922	0.273 ± 0.045	≥1.508	>3.922	0.183 ± 0.039	>3.922	>3.922
8b	>3.183	0.478 ± 0.023	>3.183	>3.183	0.230	>3.183	>3.183
8c	>2.851	0.519 ± 0.023	>2.851	>2.851	0.370 ± 0.086	>2.851	>2.851
8d	>2.623	≥1.436	>2.623	>2.623	0.736 ± 0.017	>2.623	>2.623
8e	>3.237	>3.237	>3.237	>3.237	≥1.327	>3.237	>3.237
9a	0.562 ± 0.487	0.032 ± 0.002	0.513 ± 0.415	0.903 ± 0.248	0.035 ± 0.001	1.208 ± 0.333	>3.727
9b	0.410 ± 0.350	0.103 ± 0.006	0.472 ± 0.323	>3.519	0.076 ± 0.018	>3.519	>3.519
9c	0.841 ± 0.884	0.131 ± 0.003	0.852 ± 0.655	2.250 ± 0.011	0.126 ± 0.014	≥6.136	5.874 ± 0.925
9d	0.424 ± 0.361	0.070 ± 0.025	0.428 ± 0.294	0.675 ± 0.091	0.045 ± 0.001	3.583 ± 0.241	>9.280
9e	≥4.092	0.569 ± 0.31	≥4.341	>6.687	0.642 ± 0.009	>6.687	>6.687
ETV	0.0097 ± 0.003	0.0034 ± 0.0003	0.019 ± 0.007	0.020 ± 0.0034	0.014 ± 0.0025	0.023 ± 0.011	0.026 ± 0.0041
AZT	0.0054 ±0.0004	0.0078 ± 0.0005	0.0063 ±0.0009	0.008 ±0.001	0.017 ±0.0056	0.0053 ±0.0011	0.011 ± 0.0029

## Data Availability

Data available in a publicly accessible repository.
